# The King's Fund and a Hospital Policy

**Published:** 1921-07-09

**Authors:** 


### The Hospital

The Workers' Newspaper of Administrative Medicine and Institutional Life, Administration, National Insurance and Health.

No. 1831, Vol. LXX. SATURDAY, JULY 9, 1921. PRICE TWOPENCE

### THE KING'S FUND AND A HOSPITAL POLICY.

It is now three weeks since King Edward's Hospital Fund invited representatives of the twelve teaching hospitals of London to discuss the three schemes for raising income which were advocated in paragraph 37 of the Report of Lord Cave's Committee. Beyond the facts that the meeting took place and that divergent opinions were expressed, little more has yet been heard, though the Policy Committee of the King's Fund followed up this small conference by inviting the views of all the London hospitals, and a meeting with this object is being held as we go to press. It will be recalled that Lord Cave's Committee favoured the system of weekly subscriptions, or "mass contributions" as they are now sometimes called, but drew attention to three ways of obtaining them—namely, the "Oxford" and "Sussex" schemes, both in the nature of insurance, and "organised weekly contributions" without any express insurance equivalent. In calling a conference of the teaching hospitals and inviting the opinion of the London hospitals generally, the Policy Committee of the King's Fund declared that the hospitals should accept the decision come to in the Cave Committee's Report as the basis for "immediate practical experiment." At the same time the Policy Committee recognises that the establishment of mass contributions in London is attended with difficulties not found elsewhere. Consequently the Committee is discussing, with the Hospital Saturday Fund, how best the central Funds can help to overcome them.

If the separate London hospitals possess anything like the co-operative spirit of the central Funds, something may be done to pull them out of the depression and isolation which beset them at present. We are glad to see that three hospitals—the London, St. Thomas's, and the Royal Free—announce their intention of putting one of the three schemes into practice. They have selected the Sussex scheme, known also as Dr. Gordon Dill's scheme, which was fully described in *The Hospital* of January 22 last, and have opened organising offices at 3 Fenchurch Avenue. They do not desire to monopolise the idea, but hope that by October many other hospitals will have joined them. With this lead we have no doubt other hospitals will get to work, either on the Gordon Dill scheme or one of the others, and the King's Fund can do most useful work in clearing the ground with the view of shepherding the independent London hospitals into a common policy. To this end neither the progress made nor the difficulties met should be withheld. Discussions lose much of their driving force if they cannot be read afterwards, and so made the common property of all concerned, whether or not present at the debate. For, it must be admitted, the majority of the London hospitals have still to become actively interested in the policy now proposed to them; they are hesitant and sceptical about it; their ideas are still confined to pre-war methods of appeal, *plus* contributions from patients.

With frequent iteration we have asserted our belief that mass contributions could be organised in London, that the difficulties are not insuperable, and that separate, and also united, attempts should be made to secure them. Some attempt, with some success, has been made by certain hospitals. Others, like King's College Hospital, hope that, with the help of the Saturday Fund, an autumn campaign may establish them. But in the main, though we should welcome contradiction, the London hospitals are sceptical and inert in the matter. Our own advocacy of this policy has now been enforced by the report of the Cave Committee and the action of the three London hospitals already referred to in adopting one of the alternative schemes. If a new common policy is really to be tried, every means must be taken to encourage it. We hope, therefore, that all that is being said and done will be reported, that the King's Fund Policy Committee will seek publicity for the movement which it has started, and that each "practical experiment" will be given every chance of winning support by setting out to interest every hospital officer in it. The organisation of any policy of this kind is bound to be defective if the instrument of publicity is not used, for publicity keeps alive interest in each decision and confirms belief in the reality of the effort undertaken. Without such confirmation the whole attempt has an air of unreality and academic discussion, which produces an unhappy frame of mind at the moment when mutual confidence is most desirable.

As we go to press we learn that at the meeting called by the King's Fund, held at the Mansion House on July 6, the following resolution was passed: "That this conference suggests that King Edward's Hospital Fund should co-operate with the London Regional Committee of the British Hospitals Association, the Hospital Sunday Fund, the Hospital Saturday Fund, and the League of Mercy in the organisation of local collections from employees throughout the London area." We hope to publish a report of the proceedings in our next issue.

